# Coronary Sinus Reducer: History, Current Applications, and Future Perspectives

**DOI:** 10.31083/RCM39309

**Published:** 2025-11-27

**Authors:** Fabrizio Ugo, Marco Franzino, Chiara Cavallino, Mohamed Abdirashid, Ludovica Maltese, Francesco Rametta

**Affiliations:** ^1^Cardiology Department, Sant’Andrea Hospital, 13100 Vercelli, Italy

**Keywords:** coronary sinus reducer, refractory angina, coronary artery disease

## Abstract

The coronary sinus reducer (CSR) is a percutaneous device designed to improve coronary blood flow and alleviate symptoms of refractory angina in patients with severe coronary artery disease (CAD) who are unsuitable for revascularization therapy. CSR originated from earlier surgical techniques, such as coronary sinus ligation (CSL), and functions by narrowing the coronary sinus to enhance perfusion in ischemic myocardial territories—particularly in the subendocardial regions—while also reducing microvascular resistance and increasing capillary recruitment. CSR is currently recognized as an effective treatment for patients with chronic refractory angina, especially those deemed ineligible for revascularization according to current European Society of Cardiology (ESC) guidelines. Moreover, emerging studies are expanding the understanding of the mechanism of action involved in CSR, demonstrating that this technique may also improve microvascular function, particularly in patients with coronary microvascular dysfunction. These trials have shown significant improvements in coronary microcirculation and reductions in angina symptoms, suggesting that CSR may have therapeutic potential beyond obstructive CAD. Thus, CSR may represent a promising treatment option for microvascular ischemia, thereby broadening its clinical applicability to patients with angina/ischemia and non-obstructive coronary arteries (ANOCA/INOCA).

## 1. Introduction

**Fig. 1.**
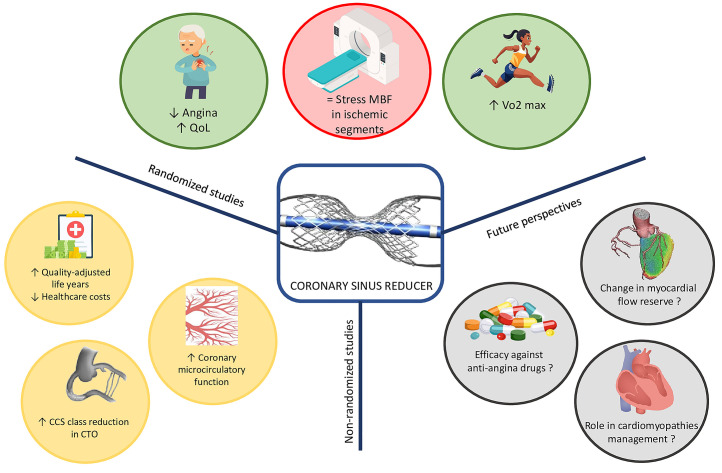
**Central figure**. The figure summarizes current knowledge on CSR derived from both randomized and non-randomized study, while also highlighting potential future research directions. CCS, Canadian Cardiovascular Society; CTO, chronic total occlusion; MBF, myocardial blood flow; QoL, quality of life.

Refractory angina remains a significant clinical challenge, especially in patients with advanced coronary artery disease (CAD) who are not candidates for revascularization. Despite the use of antianginal medications and interventions such as percutaneous or surgical revascularization, a notable proportion of CAD patients—ranging from 2% to 24%—continue to experience daily or weekly angina [1]. The coronary sinus reducer (CSR), a percutaneous device designed to modulate coronary venous outflow, has emerged as a promising therapeutic option for this patient group. Originally inspired by historical surgical techniques like coronary sinus ligation, the CSR has evolved into a minimally invasive intervention with increasing evidence supporting its safety and efficacy. This document provides a comprehensive overview of the development, mechanism of action, procedural considerations, clinical outcomes, and future prospects of CSR therapy (Fig. 1).

## 2. History

The origin of coronary sinus reducer derives from the evolution of the previous coronary sinus ligation (CSL) [2]; the first procedure involved a sternotomy and partial ligation of the coronary sinus combined with the mechanical epicardial abrasion and asbestos application to induce inflammation of pericardial surface, with the aim to stimulate collateral vessel development and promote neo-vascularization. Beck found that these operations increased collateral blood supply to ischemic myocardium beyond a ligated circumflex artery and were associated with reduced infarct size, improved myocardial contractility and reduced mortality [3, 4].

Afterwards, these surgical techniques were explored in several studies, particularly during the mid-20th century, when it was proposed as a treatment for refractory angina and heart failure. Comprehensive randomized controlled trials specifically demonstrating the efficacy of CSL are limited [5]; therefore, the lack of robust evidence and the associated risks led to its decline in favor of percutaneous techniques.

The concept of the percutaneous CSR re-emerged in the early 2000s, when Sheinfeld, Paz, and Tsehori—motivated by a growing understanding of the relationship between coronary blood flow, myocardial ischemia, and heart failure—designed a percutaneous device to emulate Beck’s surgical procedure. Banai *et al*. [6] were the first to demonstrate the efficacy and safety of CSR application in humans, paving the way for subsequent, compelling scientific research.

## 3. Current Application and Indications

In Europe, an estimated 30,000 to 50,000 new cases of chronic, debilitating, or refractory angina occur each year [7]. These patients—defined by symptoms persisting for more than three months due to reversible ischemia in the presence of obstructive CAD—are often ineligible for further medical or interventional treatment.

For this population, similar to the recommendation issued by the UK’s National Institute for Health and Care Excellence (NICE) in 2021 [8], the 2024 European Society of Cardiology (ESC) guidelines on chronic coronary syndromes recommend the implantation of a CSR to alleviate symptoms, provided the procedure is performed in experienced centers [9].

Currently, the ESC guidelines are the only international recommendations to include this technology in the management of refractory angina. However, growing evidence in recent literature may support the inclusion of CSR in future expert consensus statements and clinical guidelines on refractory angina.

## 4. Device/Procedure Characteristics

The CSR consists of a stainless-steel mesh mounted on an hourglass-shaped balloon catheter. This device is designed for percutaneous implantation into the coronary sinus (CS) to create a focal narrowing and increase coronary venous pressure. It is available in a single size that can be adjusted to the tapered anatomy of the CS by varying the balloon inflation pressure. The balloon has a diameter of 3 mm in the mid-portion and ranges from 7 mm to 13 mm at both ends.

The implantation procedure is performed percutaneously through the jugular vein, more often the right one. Initially, a 6 French (Fr) diagnostic catheter, usually a multipurpose catheter, is used to access the right atrium, where invasive central venous pressure is measured. The catheter is then used to selectively engage the CS, and contrast is injected to assess the vessel anatomy.

Despite coming from the electrophysiological field, data suggest that up to 10% of CS catheterizations fail; more often because of a Tebesian valve covering >75% of the ostium, a fibrous, fibromuscular, or muscular composition, and devoid of fenestrations that makes them a mechanical obstacle interfering with the cannulation of the CS [10].

Next, a 9 Fr guiding catheter is exchanged over a wire and positioned distally within the CS. The CSR is then advanced to the targeted implantation site, usually 1.5–3 cm distal to the CS ostium. It is important to balance the risk of device dislodgment when implanted more proximally with the lower efficacy when implanted too deeply in the coronary sinus.

Once the CSR reaches the intended location, the guiding catheter is retracted, exposing the device. The balloon is then inflated, causing the CSR to expand. After deflation, the balloon catheter is removed, and a final angiogram is performed to evaluate device positioning, confirm the narrowing, and rule out complications.

## 5. Peri-Procedural Complications

The procedure for CSR implantation is generally safe, with a high procedural success rate (98.3%) and a low incidence of complications, most of which are minor in nature.

Most recent data showed no periprocedural mortality or strokes, rare acute coronary syndrome event, all occurring days post procedure, and none directly attributed to the CSR [11, 12].

Coronary sinus dissection can occur during CSR implantation, often due to damage from catheter or wire manipulation against the delicate coronary sinus wall or as a result of pneumatic damage following contrast injection. While coronary sinus dissections rarely progress to complete perforation, they can usually be managed conservatively, with prolonged balloon inflation and with the implantation of the CSR in the true lumen, which typically seals the dissection and resolves the complication.

A more severe complication is coronary sinus perforation, which may result from excessive oversizing during scaffold deployment, distal migration of the wire tip (especially into small side branches), scaffold deployment partially within a side branch due to wire migration or brisk contrast injection to a small branch.

Management of coronary sinus perforation depends on the severity, location, and hemodynamic compromise. The initial step involves stopping the bleeding by inflating a balloon at the perforation site or distally (confirming resolution of the extravasation through contrast injection) and performing pericardiocentesis in cases of cardiac tamponade (Table 1). While most perforations resolve without further intervention, more severe cases may have persistent extravasation despite prolonged balloon inflation, requiring the placement of polytetrafluoroethylene (PTFE) stents or, in some cases, emergency cardiac surgery [13, 14, 15, 16]. A recent meta-analysis showed a coronary sinus perforation rate of 0.008% and a cardiac tamponade rate of 0.001% [11].

**Table 1.** **Complications, preventive strategies and treatment**.

Complication		Preventive strategies	Treatment
Dissection		Handle the device gently; don’t push the wire against resistance; check blood backflow before contrast injection	1. Balloon inflation;
			2. CSR implantatiom.
Perforation			
	Main vessel	Precisely sizing of CSR; check wire position; check catheter position and blood back flow before contrast injection	1. Prolonged balloon inflation and pericardiocentesis (if cardiac tamponade);
			2. Covered stent implantation;
			3. Cardiac surgery.
	Distal vessel	Check wire position; check blood backflow before contrast injection; don’t use polymeric wires	1. Prolonged balloon inflation and pericardiocentesis (if cardiac tamponade);
			2. Cardiac surgery.
Scaffold migration			
	Partially overlapping the balloon	Handle the device gently	1. Reducer balloon inflation (to deploy the scaffold partially);
			2. Advance and deploy a second balloon to expand the distal part of the device;
			3. If 2 ineffective fully expand it and lose the narrow neck;
			4. Implant another CSR inside.
	Uncrimped device not overlapping the balloon	Handle the device gently	1. Snare the scaffold with Gooseneck snares;
			2. Advance a small balloon beyond the device and inflate trying to retrieve the scaffold;
			3. Advance a balloon inside the scaffold inflated to expand the device to the vessel wall (progressively larger sized balloons are needed);
			4. Implant another CSR inside.
	Scaffold migration after deployment	Inflate the balloon without exceeding a 20%:80% contrast/saline ratio saline or even without contrast; perform several balloon deflations waiting for the full deployment	1. Gently push the guiding catheter against its neck to re-advance the scaffold in the correct position;
			2. Advance a second 0.035” wire through the device mesh at the neck level and push a 5 Fr diagnostic multipurpose (MP) catheter, inserted over the 2° wire, against the neck to advance the scaffold in the correct position.
Guide catheter entrapment into the device’s neck			
	Catheter tip inside the CRS	Inflate the balloon without exceeding a 20%:80% contrast/saline ratio saline or even without contrast; perform several balloon deflations waiting for the full deployment	1. Gently push the wire trying to disengage the catheter;
			2. Advance a second balloon over the wire, inflate it at the distal part of the device (trapping it) and gently pull back the guiding catheter.
	Catheter tip beyond the CSR	Inflate the balloon without exceeding a 20%:80% contrast/saline ratio saline or even without contrast; perform several balloon deflations waiting for the full deployment	1. Advance a larger catheter against the scaffold neck over the 9 Fr guiding catheter (“mother and child” technique) and gently pull back the guiding catheter;
			2. Advance a second guide catheter and a 0.035” wire through the scaffold struts at the level of the neck, push the second catheter against the neck and gently pull back and detach from the scaffold the first guiding catheter.

CSR, coronary sinus reducer; Fr, French.

Balloon retrieval following CSR deployment must be performed carefully, as the large profile of the deflated balloon can lead to several complications, including proximal displacement of the device, device deformation, and catheter tip entrapment within the narrowed center. These complications can be prevented by inflating the balloon without exceeding a 20% : 80% contrast/saline ratio or even without contrast inside, avoiding inflation pressures greater than 6 atmospheres, performing several deflations of the balloon before retrieval, and gently pulling the balloon into the guiding catheter that has been positioned against the narrow neck of the scaffold.

Scaffold migration, the most common complication (occurring in approximately 1.5% of recipients overall), may occur during different phases of the procedure [11]. In contrast, device deformation and catheter tip entrapment are frequently caused by balloon retrieval. Depending on the setting, several strategies can be performed to solve the complication, as summarized in Table 1 [13, 17].

## 6. Mechanism of Action

The CSR device, when implanted within the CS, reduces its lumen size to a fixed diameter of 3 mm, thereby increasing the CS pressure.

Under physiological conditions, during exercise, sympathetic-mediated vasoconstriction of the sub-epicardial vessels increases blood flow to the sub-endocardial layers [18]. However, this mechanism becomes impaired in the presence of obstructive CAD, and the elevated left ventricular end-diastolic pressure often seen in ischemic heart disease can directly affect myocardial wall kinetics. This results in increased coronary flow resistance and sub-endocardial ischemia.

By inducing a focal narrowing of the CS in the setting of ischemic myocardium, the CSR raises venous pressure within the coronary venous system. This leads to the dilation of arterial capillaries and a reduction in flow resistance, facilitating the redistribution of blood from the relatively less ischemic sub-epicardium to the more ischemic sub-endocardium [19, 20]. The beneficial effects of CSR are typically observed once the device has undergone endothelialization, which usually occurs within 1–3 months after implantation.

A recent simulation study suggests that the mechanisms of action of the CSR vary depending on the severity of coronary artery stenosis [21]. In cases of moderate stenosis, the primary benefit of CSR is an increase in coronary transit time (CTT), which helps improve tissue oxygenation. Differently, with severe stenosis, the main advantages of CSR are the redistribution of blood from non-ischemic to ischemic regions and a reduction in capillary flow heterogeneity [21].

All the mechanisms described—those that enhance myocardial perfusion, improve the endocardial-to-epicardial reserve index, and reduce ischemic burden—appear to be effective not only in epicardial obstructive disease but also in microvascular disease [22].

Indeed, a randomized clinical trial showed that an acute increase in coronary sinus pressure in patients with angina and microvascular dysfunction resulted in a significant reduction in microvascular resistance and an increase in blood flow [23]. A Phase II clinical trial assessing the feasibility and potential efficacy of CSR implantation for coronary microvascular dysfunction demonstrated that the device significantly improved angina symptoms, quality of life, and coronary microvascular function with an improvement in both endothelium-dependent and endothelium-independent microvascular function [24].

This evidence suggests that the effects of CSR are not uniform, but rather adapt to the severity and type of underlying coronary pathology—whether obstructive or non-obstructive—highlighting its role in optimizing coronary perfusion based on individual patient characteristics.

## 7. Current Knowledge of Efficacy and Safety

Several studies were developed to demonstrate the efficacy and safety of CSR (Table 2, Ref. [6, 18, 19, 24, 25, 26, 27, 28, 29, 30, 31, 32, 33, 34, 35, 36, 37, 38, 39, 40]).

**Table 2.** **Published studies of coronary sinus reducer**.

Study	Year	Study design	Population	Outcome	Follow-up
Banai *et al*. [6]	2007	Non-randomized, open-label, prospective	15	CSR was found to be safe and feasible	11 months
Konigstein *et al*. [34]	2014	Prospective	23	Angina score expressed by CCS class decreased significantly (85% of patients reported relief of symptoms)	11 months
Verheye *et al*. [19]	2015	Randomized, double-blind, sham-controlled	104	A reduction of at least 2 CCS classes was found 2.4 times more frequently in the CSR group	6 months
Giannini *et al*. [31]	2020	Non-randomized, prospective cohort	8	Median CCS class improved from 3.0 to 1.5 in CSR group (*p* = 0.014)	6 months
Konigstein *et al*. [18]	2018	Non-randomized, prospective, open-label registry	48	(1) CCS class diminished from a mean of 3.4 ± 0.5 at baseline to 2.0 ± 1 (*p * < 0.001)	12.5 months
				(2) Ejection fraction (EF%) at stress increased from 51.0 ± 10 to 56.5 ± 10 (*p* = 0.004)	
Giannini *et al*. [32]	2018	Observational, single center	50	(1) At least 1 CCS class reduction in 80% of patients	4 and 12 months
				(2) At least 2 class reduction in 40% of patients	
				(3) Significant quality of life improvement, improvement in 6-minute walking test, reduction in the requirement of anti-anginal drug treatment	
Giannini *et al*. [35]	2017	Multicenter, retrospective, observational	215	CSR was associated with higher QALYs and incremental costs, yielding ICERs	1 year
Tzanis *et al*. [36]	2020	Prospective	19	(1) Significant improvement in LVEF was found after CSR	4 months
				(2) Angina class significantly improved after CSR (84% at least one CCS class)	
Zivelonghi *et al*. [28]	2020	Retrospective	205	Significantly higher improvement in CCS class in the CTO-group	6 months
Verheye *et al*. [25]	2021	Non-randomized, prospective with a retrospective component, open-label, two-arm observational study	228	An improvement of ≥1 CCS class was recorded in 74% of patients at 1 year, and in 82% of patients at 2 years	2 years
Jolicoeur *et al*. [37]	2021	COSIRA post hoc analysis	104	Improvement of symptoms, functionality and quality of life among patients treated with CSR	6 months
Silvis *et al*. [38]	2021	Prospective cohort	132	Improvement of at least 2 CCS class (34%)	6 months
Konigstein *et al*. [33]	2021	Multicenter, prospective cohort	99	Improvement of CCS class to 1.66 ± 0.8 at 1 year (*p* < 0.001), and remained low through 2-years and at last follow up (1.72 ± 0.8 and 1.71 ± 0.8, *p* > 0.5 for both, in comparison to 1 year).	3.38 years
D’Amico *et al*. [39]	2021	Non-randomized, prospective cohort	187	Reduction of at least 2 CCS classes was observed in 49% of patients	18.4 months
Mrak *et al*. [29]	2022	Non-randomized, prospective cohort	22	Mean CCS class improved in CTO RCA group from 2.73 to 1.82 (*p * < 0.001)	12 months
Mrak *et al*. [30]	2023	Randomized, double-blinded, sham-controlled	25	Maximal oxygen consumption increased from 15.56 ± 4.05 to 18.4 ± 5.2 mL/kg/min (*p* = 0.03) in CSR group but did not change in the sham group (*p* = 0.53)	6 months
Foley *et al*. [26]	2024	Randomized, double-blind, placebo-controlled	61	(1) Myocardial blood flow in ischaemic segments did not improve with CSR compared with placebo.	6 months
				(2) The number of daily angina episodes was significantly reduced with CSR compared with placebo.	
Tebaldi *et al*. [27]	2024	Multicenter, prospective, single-cohort, investigator-driven	24	The IMR values decreased from 33.35 ± 19.88 at baseline to 15.42 ± 11.36 at 4-month follow-up (*p * < 0.001; mean difference, −17.90)	4 months
Verheye *et al*. [40]	2024	Observational	400	An improvement of ≥1 CCS class was recorded in 68.9% of patients	6 months
Tryon *et al*. [24]	2024	Phase II trial	20	In patients with endothelium-independent CMD CFR increased significantly from 2.1 (1.95–2.30) to 2.7 (2.45–2.95) (*p* = 0.0011). In patients with endothelium-dependent CMD had an increase in CBF response to acetylcholine (*p* = 0.042)	4 months

CCS, Canadian Cardiovascular Society; CSR, coronary sinus reducer; ICERs, incremental cost-effectiveness ratios; IMR, Index of microcirculatory resistance; LVEF, left ventricular ejection fraction; QALYs, quality-adjusted life years; CTO, chronic total occlusion; CFR, coronary flow reserve; CMD, coronary microvascular dysfunction; CBF, coronary blood flow; RCA, right coronary artery.

The COSIRA trial, a multicenter, randomized, double-blind, sham-controlled clinical study, enrolled 104 patients with Canadian Cardiovascular Society (CCS) class III or IV angina randomly assigned to receive either the device or a sham procedure. At 6 months, 35% of the treatment group had an improvement of at least two CCS angina classes, compared to 15% in the control group. The treatment group also showed significant improvement in quality of life, as measured by the Seattle Angina Questionnaire [19].

The REDUCER-I trial, a multicenter, non-randomized observational study enrolled patients with refractory angina (CCS class II–IV) treated with Reducer implantation; demonstrating the high safety profile of this therapy and the sustained improvement in angina severity and in quality of life up to two years [25]. More recently, the ORBITA-COSMIC, a double-blind, randomized, placebo-controlled trial conducted in the UK, involving 61 patients who were randomly assigned to either the CSR or placebo group showed no significant improvement in myocardial blood flow with CSR compared to placebo; however, patients receiving CSR experienced a reduction in daily angina episodes compared to those in the placebo group, suggesting that CSR may be effective in improving symptoms of angina. Importantly, there were no deaths or acute coronary events in either group, though two cases of CSR embolization occurred in the CSR group [26].

Even three meta-analyses demonstrated the promising efficacy of CSR in refractory angina: the first, including eight registries and one randomized controlled trial (RCT) involving a total of 846 patients with refractory angina, found that the use of a coronary sinus reducer resulted in an improvement of at least one CCS class in 76% of patients and an improvement of at least two CCS classes in 40% of patients [41]. The second included 10 studies with a total of 799 patients and demonstrated a significant improvement in all outcomes analyzed: the CCS classification, the Seattle Angina Questionnaire (SAQ) score and the six-minute walk distance (6MWD) [42]. The third included 13 studies with a total of 848 patients, demonstrating that placebo-controlled rates were 26% (95% CI: 11%–38%; *p* < 0.001) for ≥1-class CCS improvement and 17% (95% CI: 2%–37%; *p* = 0.02) for ≥2-class CCS improvement [11].

The next level of evidence was driven by INROAD trial, a prospective, multicenter, single-cohort, investigator-driven study that involved 24 patients with a history of obstructive coronary artery disease and prior revascularization underwent invasive physiological assessments before and 4 months after the CSR showing a significant enhanced of coronary microcirculatory function and a reduction in angina symptoms, suggesting its potential as a treatment for coronary microvascular dysfunction [27].

In the setting of chronic total occlusion coronary artery, Zivelonghi *et al*. [28] demonstrated that patients with refractory angina and non-revascularized chronic total occlusion (CTO) lesions had a better response to CSR implantation than those without CTOs; suggesting that CSR implantation can be considered a valuable complementary therapy for patients with CTO lesions, potentially enhancing outcomes when PCI for CTO is not feasible or successful. Mrak *et al*. [29] have consolidated this aspect showing that CSR implantation in patients with refractory angina due to CTO of the right coronary and predominant inferior and inferoseptal left ventricular wall ischemia improves angina symptoms and QoL.

Mrak *et al*. [43] have also theorized the possibility that CSR might have an antiarrhythmic effect, however they did not demonstrate a significant impact on the arrhythmogenic substrate assessed with high-resolution electrocardiogram (hrECG) parameters. But the most interesting finding of Mrak *et al*. [30] is that CSR maximal oxygen consumption during the cardiopulmonary exercise test significantly increases and doesn’t change in the sham group.

Regarding the cost-effectiveness of CSR, Gallone *et al*. [31] showed a significant reduction in hospitalizations, outpatient visits, and interventions in patients treated with CSR, translating into lower costs and higher quality-adjusted life years. Cost-effectiveness analysis showed that CSR was cost-effective across Belgium, the Netherlands, and Italy, with favorable incremental cost-effectiveness ratios (ICERs) [31].

Furthermore, as conceivable, Giannini *et al*. [32], in addition to demonstrating that CSR reduces CCS class and improves 6-minute walk distance, showed a decrease in antianginal drug use at one-year follow-up, with respective reductions of 32% and 6% in at least 1 or 2 antianginal medications.

## 8. Future Perspective 

Considering the current knowledge about the efficacy of CSR in reducing angina episodes and improving microvascular dysfunction, further investigation into its application in cardiomyopathy-related angina would be desirable. This includes conditions such as hypertrophic cardiomyopathy and its phenocopies, where myocardial disarray and microvascular disease play a significant role.

Another interesting area for exploration is CTO coronary artery disease. For patients with CTO who are unsuitable for percutaneous coronary intervention (PCI) and experience recurrent angina episodes, CSR treatment should be considered in future trials.

It would also be tempting to explore the effectiveness and cost-effectiveness of CSR compared to antianginal drugs, which are often not free from side effects.

Ongoing randomized studies are exploring new areas to address the gap in CSR efficacy and safety (Table 3). The COSIRA-II study is a multicenter, randomized, double-blinded, sham-controlled clinical trial that aims to evaluate changes in total exercise duration using the modified Bruce treadmill exercise tolerance test in patients with refractory angina pectoris. Patients enrolled must demonstrate objective evidence of reversible myocardial ischemia in the distribution of the left coronary artery, which is unsuitable for revascularization. Notably, two imaging sub-studies (using positron emission tomography and computed tomography) in a non-randomized arm will provide additional insights into safety and the mechanism of action of CSR.

**Table 3.** **Ongoing studies**.

Study	Study design	Population	Primary outcome	Time frame
COSIRA-II (NCT05102019)	Multicenter, randomized, double-blinded, sham-controlled	380	Total exercise duration using the modified Bruce treadmill exercise tolerance test	6 months
REMEDY-PILOT, REMEDY-MECH (NCT05492110)	Randomized, double-blinded, sham-controlled	54	Quantitative myocardial perfusion (global myocardial perfusion reserve [MPR]) assessed by cardiac MRI.	6 months
COSIMA (NCT04606459)	Multicenter, randomized	144	Change in CCS angina class	6 months
MICS-Reduce (NCT06033495)	Observational, prospective	15	Change in myocardial flow reserve on ^15^O-H_2_O PET/CT	6 months
NCT04523168	Single center, prospective, single arm	30	Change in CFR	120 days
NCT01566175	Multicenter, prospective, single arm	100	Change in CCS angina class	6 months

CCS, Canadian Cardiovascular Society; CFR, coronary flow reserve; CT, computed tomography; MPR, myocardial perfusion reserve; MRI, magnetic resonance imaging; PET, positron emission tomography.

Finally, the REMEDY-PILOT and COSIMA randomized trials, aim to investigate the applicability of CSR in populations with ischemia and non-obstructive coronary artery disease, including patients with coronary microvascular dysfunction.

## 9. Conclusion

Today, the Coronary Sinus Reducer is considered an effective and safe treatment for reducing angina and improving quality of life in patients with refractory angina. This includes those with epicardial or microvascular coronary artery disease who are not candidates for further medical or interventional treatments. Ongoing studies seek to broaden the current understanding and extend the therapeutic uses of CSR to a wider range of patients.

## Abbreviations

CAD, coronary artery disease; CCS, Canadian Cardiovascular Society; CSL, coronary sinus ligation; CSR, coronary sinus reducer; CTO, chronic total occlusion; ESC, European Society of Cardiology; PCI, percutaneous coronary intervention; RCT, randomized controlled trial; SAQ, Seattle Angina Questionnaire; 6MWT, six-minute walk distance; RCA, right coronary artery.
